# Exploring the potential of AI-driven food waste management strategies used in the hospitality industry for application in household settings

**DOI:** 10.3389/frai.2024.1429477

**Published:** 2025-01-23

**Authors:** Quintana M. Clark, Disha Basavaraja Kanavikar, Jason Clark, Patrick J. Donnelly

**Affiliations:** ^1^Department of Agricultural Sciences Education and the Department of Educational Practice and Research, Oregon State University, Corvallis, OR, United States; ^2^Department of Electrical Engineering and Computer Science, Science, Oregon State University Cascades Campus, Bend, OR, United States

**Keywords:** AI, food waste, sustainable technology, AI-driven, households

## Abstract

This study explores the potential for adapting AI-driven food waste management strategies from the hospitality industry for application in household settings. The hospitality industry, particularly hotels and restaurants, has implemented AI technologies through companies like Leanpath, Winnow, and Kitro, which use real-time data and predictive analytics to monitor, categorize, and reduce food waste. These AI-driven systems have demonstrated significant reductions in food waste, offering economic savings and environmental benefits. This study employs an instrumental case study approach, utilizing semi-structured interviews with representatives from these companies to gain insights into the technologies and strategies that have proven effective in hospitality. The findings suggest that with modifications for scale, cost, and user engagement, AI-driven solutions could enhance household food management by providing insights into consumption patterns, offering expiration reminders, and supporting sustainable practices. Highlighted are key considerations for household adaptation, including policy support, educational strategies, economic incentives, and integration with smart home systems. Ultimately, this study identifies a promising avenue for reducing household food waste through AI, underscoring the need for continued research and policy initiatives to facilitate the transition of these technologies from commercial kitchens to everyday homes.

## 1 Introduction

Food waste is a critical global issue, with nearly one-third (about 2 billion tons) of all food produced annually for consumption going to waste (Xue et al., 2017; Fang et al., 2023). This wastage leads to approximately 3 billion tons of greenhouse gases being released from landfills and an annual economic loss of nearly $160 billion US dollars (Yu and Jaenicke, 2020). Food waste’s environmental and economic consequences are significant, representing a lost economic opportunity and negative ecological impacts. For example, when wasted food is deposited in landfills, it generates methane gas as it decomposes. Methane is more harmful to global warming than carbon dioxide (Xue et al., 2017; Fang et al., 2023). Additionally, food waste strains the agricultural industry, consuming vast amounts of valuable resources such as water, land, and energy for food that ultimately goes unconsumed (Kummu et al., 2012; Papargyropoulou et al., 2014).

The hospitality industry, which includes hotels, restaurants, and catering services, is particularly prone to food waste due to its operational nature (Papargyropoulou et al., 2019; Dhir et al., 2020). This sector operates on a large scale, with daily food preparation and consumption occurring in high volumes. The unpredictable nature of consumer demand and operational practices such as buffet-style dining and strict food safety regulations often result in a significant surplus of food that must be discarded (Papargyropoulou et al., 2019; Dhir et al., 2020). Furthermore, over-preparation in restaurants, where chefs prepare more food than necessary to avoid running out of popular dishes during peak hours, and plate waste from customers, where uneaten food is left on plates, contribute to the overall waste (Parfitt et al., 2010). This waste includes leftover food and the resources used in its production, such as water, energy, and labor (Kummu et al., 2012; Dhir et al., 2020). The disposal of this waste typically involves transporting it to landfills, where it decomposes and releases methane gas, exacerbating global warming (Fang et al., 2023; Papargyropoulou et al., 2014).

According to the Food Waste Index Report published by the United Nations Environment Program in 2021, 61% of total food waste occurs in households, 26% in food service establishments, and 13% in retail businesses (Xue et al., 2017; United Nations Environment Programme, 2021). These statistics highlight the urgent need to reduce food waste at the hospitality and household levels. Given the magnitude of the issue, there has been a push for innovative solutions to address food waste, particularly in industries where waste is most prevalent (Marvin et al., 2022; Papargyropoulou et al., 2014). Leading companies in the hospitality industry, such as Leanpath (Food Waste Intelligence, 2024), Winnow (The Food Waste Blog, 2024), and Kitro (2024), have risen to the challenge by leveraging artificial intelligence (AI) to address food waste. These companies have developed AI-driven technologies integrating with existing kitchen operations to monitor and analyze food usage in real-time. By tracking the quantity and type of wasted food, AI systems provide insights that help kitchen staff adjust portion sizes, optimize inventory management, and reduce over-preparation (Marvin et al., 2022). By identifying patterns in food waste, these technologies enable implementing more sustainable practices, such as menu adjustments and more accurate demand forecasting, ultimately reducing waste and its associated environmental impact (Fang et al., 2023; Marvin et al., 2022; Dhir et al., 2020).

However, while significant strides have been made in reducing food waste at the hospitality level, food waste management using AI-driven technology at the household level remains an area that needs further exploration (Dhir et al., 2020; Parfitt et al., 2010). Through interviews with major hospitality industry companies, the objective of this study seeks insights into how these innovative solutions can be adapted for household use (Creswell and Poth, 2016). As households contribute significantly to food waste, adopting these methods could benefit policymakers and consumers alike (Janssens et al., 2019; Teng et al., 2021).

This paper is organized as follows: Section 2 provides a background of food waste and discusses AI’s role in food waste management within the hospitality industry and its potential applicability in the home environment. Section 3 explores theoretical and managerial perspectives on the potential for adapting AI-driven solutions in household food loss and waste management. Section 4 outlines the methods and procedures used in this case study. Section 5 presents the findings from interviews with Leanpath (Food Waste Intelligence, 2024), Winnow (The Food Waste Blog, 2024), and Kitro (Kitro, 2024). Section 6, the discussion section, explores how AI-driven food waste management strategies might be adapted for household use. Section 7 discusses potential implications for household adaptation, policy recommendations, and strategies for research and education. Section 8 addresses the limitations of the study. Finally, Section 9 concludes.

## 2 Background

Food waste management refers to the practices and strategies implemented to reduce food waste in both post-production and consumption (Sustainable Management of Food Basic, 2023). This process minimizes food waste’s negative environmental, economic, and social impacts. Food waste management encompasses a range of activities, from preventive measures that reduce food waste at the source to AI-driven methods for recovering and recycling food waste, such as returning nutrients to the soil through composting, greenhouse gas production from landfills, redistribution to those in need through food banks, or creating renewable energy (Sustainable Management of Food Basic, 2023). Effective food waste management not only aids in environmental conservation but also can result in cost savings and resource optimization.

The global concern surrounding food waste has risen over the past few decades. Research indicates that nearly a third of all food produced for human consumption gets lost or wasted, which equates to approximately 1.3 billion tons annually (Xue et al., 2017; United Nations Environment Programme, 2021). Such vast amounts of unconstrained food waste have dire consequences, including exacerbating greenhouse gas emissions from landfills, significant financial losses, and an increased strain on the agricultural industry (Fang et al., 2023). The paradigm of “from farm to fork” has expanded to “from farm to fork to landfill,” illuminating the gaps in our current food systems (Gunders and Bloom, 2012). Urban food waste management becomes vital as urban areas grow and population densities increase. Efficiently managing this waste can significantly curb the negative environmental impacts and promote sustainable living (Kaza et al., 2018).

Household food waste remains one of the most overlooked yet impactful areas of waste generation (Bunditsakulchai and Liu, 2021). Households must purchase wisely, store efficiently, and consume prudently to minimize waste (Bunditsakulchai and Liu, 2021). Composting serves as a recycling mechanism, turning organic waste into a resource for gardening. Despite these practices, a significant volume of food remains in landfills, with unconstrained waste contributing to greenhouse gas emissions, financial loss, and undue pressure on the agricultural industry.

### 2.1 AI-driven technology in the hospitality industry

The digital age has seen AI permeate various industries, revolutionizing operations and business practices (Fang et al., 2023). The hospitality industry, grappling with substantial food waste, has been no exception (Papargyropoulou et al., 2019). AI’s data-driven approach offers predictive analytics, automates waste management, and provides actionable insights to reduce food waste significantly (Fang et al., 2023; Marvin et al., 2022). Innovations in AI-driven technology have been introduced to tackle the issue of food waste, especially in industries where the volume of waste is exceptionally high. The hospitality industry, a significant contributor to the problem, has adopted various tech-driven solutions to optimize operations and reduce food waste (Engström and Carlsson-Kanyama, 2004). The hospitality industry, comprising hotels, restaurants, and catering services, is pivotal in global food distribution and consumption. Historically, this industry has been identified as a significant contributor to food waste due to the industry’s operational nature, which necessitates surplus provisioning to meet diverse consumer demands. With high standards for food presentation and quality, substantial amounts often get discarded. This waste not only implies economic losses but also an unnecessary environmental footprint. The hospitality industry has realized that efficient food waste management can bring tangible economic benefits beyond the apparent environmental and moral imperatives. As a result, there is an increasing trend in the industry to employ advanced AI-driven technologies to devise and implement effective waste management strategies (Fang et al., 2023; Papargyropoulou et al., 2014).

In recent years, several technological solutions have been developed to address the challenge of food waste in the hospitality industry. Leanpath, Winnow, and Kitro have emerged as leaders in leveraging AI to mitigate waste. Their systems employ sophisticated algorithms and sensors to identify, categorize, and quantify discarded food. These real-time insights provide actionable feedback to the hospitality industry, enabling them to adjust their practices and reduce food waste. The success of such AI-driven systems in a high-waste environment like hospitality sparks interest in their potential applicability to the household environment. The advancements from these organizations have led to a paradigm shift in how the hospitality industry approaches food waste. These AI systems facilitate immediate intervention and longer-term strategic planning by offering real-time analytics. The cumulative effect of these technologies is not just limited to economic savings for businesses. They have contributed to a more sustainable and responsible hospitality industry, setting the stage for scalable solutions that might be transferred to the home environment (Dhir et al., 2020).

### 2.2 AI’s potential applicability to home environment

There is potential for adapting AI-driven solutions from the hospitality industry for household use. While households represent a significant contributor to food waste, their inconsistent and varied food consumption patterns pose challenges to applying AI innovations directly. However, with the advent of smart home systems and the Internet of Things (IoT), there is an opportunity to utilize predictive analytics for improved food consumption, purchasing decisions, and waste reduction (Fang et al., 2023). Through user-friendly applications, households can optimize food utilization, providing economic and environmental benefits. The success of AI-driven interventions in the hospitality industry poses a pertinent question, i.e., what is the potential applicability of AI-driven food waste management methods from the hospitality industry to the household environment?

Households form the base unit of food consumption, collectively representing a significant proportion of food waste (Parfitt et al., 2010). Addressing the issue at this micro-level can exponentially enhance the larger objective of curbing food waste at the macro level. Several challenges must be considered when translating the large-scale practices of the hospitality industry to individual households. For instance, food waste volume and consistency in restaurants and hotels are often more predictable than in domestic settings, where variability in consumption patterns, dietary choices, and purchasing behaviors come into play (Janssens et al., 2019). The economic incentives for households to invest in advanced AI-driven solutions may not be as immediately apparent as they are for businesses. Nevertheless, there are many potential advantages. With the integration of AI into smart home systems and IoT devices, households can benefit from predictive analytics. Such systems could offer real-time insights into consumption patterns, recommend grocery purchasing decisions based on historical data, and provide creative solutions for repurposing or composting leftover food. All of these assist in reducing food waste and can contribute to significant savings for households in the long run (Teng et al., 2021).

Table 1 provides a side-by-side comparison of AI-driven food waste management features as applied in the hospitality industry versus potential adaptations for household use. Each feature illustrates how these technologies, originally designed for large-scale, high-volume settings, can be scaled down and customized to address the unique needs of individual households. In hospitality settings, *Smart bins with scale and AI camera* automatically track, weigh, and categorize food waste, helping commercial kitchens monitor waste and make data-driven adjustments (Fang et al., 2023; Marvin et al., 2022; Bux, 2024; Bux and Amicarelli, 2024). In a household context, a more compact version of this bin would track waste types and amounts, offering simplified insights. *Centralized dashboard monitoring* (Bux, 2024; Bux and Amicarelli, 2024) is another key feature in hospitality, where a large display provides real-time waste metrics to staff. At home, a mobile app could serve as a personal dashboard, summarizing waste data, notifying users about expiring items, and offering waste reduction tips. *Predictive inventory management* (Bux, 2024; Bux and Amicarelli, 2024) helps commercial kitchens adjust stock levels based on historical demand, while a household version might be integrated into a smart fridge to remind users of upcoming expiration dates. Additionally, *AI-enhanced point of sale (POS) systems* (Bux, 2024; Bux and Amicarelli, 2024) optimize portion sizes and menu planning in hospitality; for households, a mobile app could suggest weekly shopping lists or meal plans based on past consumption patterns to avoid overbuying. Lastly, *data-driven feedback loops* and *employee training & engagement* (Bux, 2024; Bux and Amicarelli, 2024) in hospitality encourage continuous improvements and participation, while at the household level, app notifications can remind users to use leftovers and suggest recipes, fostering mindful consumption. Table 1 highlights both the adaptability and potential barriers to transferring these advanced technologies into everyday household environments.

**Table 1:** Comparison of AI-driven food waste management features in hospitality vs. household settings.

Feature	Hospitality setting	Household setting
Smart bin with scale and AI camera	Large commercial smart bins with integrated cameras and scales for precise food waste tracking, identifying food types, and weighing discarded items. Data is used for real-time analytics.	Compact smart bin with a basic camera and scale to track and weigh household food waste. Provides simplified data on waste amounts and types.
Centralized dashboard monitoring	Real-time monitoring on a centralized dashboard in the kitchen, providing detailed analytics on food waste sources, types, and costs.	Mobile app dashboard providing a weekly or daily summary of food waste data, notifications for expiring items, and tips for reducing waste.
Predictive inventory management	AI-driven inventory system linked to suppliers, predicting stock needs based on historical data to avoid over-ordering and minimize waste.	Smart fridge with AI capabilities, tracking food items inside and offering shopping reminders or suggestions to use soon-to-expire items.
AI-enhanced point of sale (POS) system	POS system integrated with AI to collect data on popular menu items, adjusting portion sizes, and minimizing over-preparation.	Shopping and meal planning suggestions via the mobile app, based on previous household usage patterns, to help prevent overbuying.
Data-driven feedback loops	Automated feedback sent to kitchen staff, helping them adjust portion sizes, menu offerings, and inventory levels.	Notifications on the mobile app reminding users to use leftovers or suggesting recipes based on available ingredients.
Employee training and engagement	Staff receive training and ongoing feedback on food waste practices, often with features like leaderboards or waste reduction goals to encourage participation.	Home users receive waste reduction tips and personalized suggestions on the app, encouraging mindful consumption habits.

This table compares the adaptation of AI-driven food waste management features from large-scale hospitality settings to individual household environments. While hospitality applications are optimized for high-volume operations, household adaptations focus on user-friendly, compact solutions with simplified data outputs.

## 3 Literature review of theoretical and managerial perspectives

Household food loss and waste are significant contributors to global food waste, posing challenges to sustainability, food security, and environmental conservation (Kitro, 2024; Bux and Amicarelli, 2024). Recent developments in AI-driven solutions have shown the potential to mitigate this issue by leveraging predictive technologies and data-driven insights to manage food consumption and waste. This literature review explores theoretical and managerial perspectives on the potential for adapting AI-driven solutions, developed initially for sectors such as hospitality, to manage household food loss and waste. The behavioral and technological theories discussed provide a framework for understanding how AI can influence household decision-making, behavior, and waste management reduction practices.

*Theory of planned behavior (TPB)*: Ajzen (1991) Theory of Planned Behavior (TPB) suggests that behavior is driven by three primary factors: attitudes, subjective norms, and perceived behavioral control. In the context of food waste, TPB can explain how AI-driven tools can positively influence consumer behavior. AI systems that provide timely reminders, consumption data, or personalized recommendations enhance individuals’ perceived control over food management, reducing waste (Stancu et al., 2016). For instance, smart refrigerators and AI-powered apps track food expiration dates and suggest meal plans, thus reinforcing responsible food consumption behaviors. When integrated into household settings, these AI-driven tools can shift attitudes toward reducing waste by making the process more convenient and accessible (Roodhuyzen et al., 2017). *Nudge theory*: Nudge Theory, popularized by Thaler and Sunstein (2008), posits that small environmental changes can encourage better decisions without restricting freedom of choice. AI systems in food waste management apply nudges by sending notifications to users about food approaching expiration or suggesting recipes to use up leftovers. In commercial settings like hospitality, nudges have been implemented to reduce waste by adjusting portions and optimizing inventory (Principato et al., 2021).

When adapted to households, AI-driven nudges can be equally effective. Applications like OLIO (Olio, 2024) and Too Good To Go (Too Good To Go, 2024) use notifications to nudge consumers into donating excess food or redistributing surplus before it becomes waste. However, their effectiveness in households depends heavily on sustained user engagement, as occasional use may not be enough to drive long-term behavioral change (Filimonau and Gherbin, 2017). *Rational choice theory*: Rational Choice Theory (Becker, 1976) assumes that individuals make decisions by evaluating costs and benefits. AI systems align with this theory by reducing the cognitive load required to make food management decisions. By automating tasks such as inventory tracking, meal planning, and expiration date alerts, AI tools offer households rational solutions to minimize waste (Aschemann-Witzel et al., 2015). In the hospitality industry, AI technologies optimize supply chains and reduce waste by providing real-time data on inventory levels. Similar household tools can help consumers make more informed purchasing decisions, thus reducing the likelihood of overbuying and food spoilage (Borrello et al., 2016). However, the success of such AI tools depends on whether households perceive the long-term benefits as outweighing the initial costs and effort involved in adopting the technology (Quested et al., 2013). *AI and machine learning frameworks*: AI technologies use machine learning algorithms to predict food consumption patterns and provide personalized recommendations. Predictive analytics, a common AI technique, uses historical data to forecast future food usage, helping households reduce overbuying and better manage their stock (van der Werf et al., 2021). The ability of AI systems to learn from user behavior and adjust their recommendations over time is particularly valuable in household settings where consumption patterns can be irregular. In the hospitality industry, predictive analytics has significantly reduced food waste by up to 30% in some settings (Filimonau and Gherbin, 2017). However, adapting such models to households presents challenges due to the variability in household consumption patterns and the often-limited data available. Nonetheless, the potential for AI to provide adaptive and personalized solutions remains promising (Roodhuyzen et al., 2017).

From a managerial standpoint, adopting AI-driven solutions for household food waste management requires considering several factors: cost, technological infrastructure, consumer trust, and long-term engagement. Lessons from the hospitality industry, where AI-driven solutions are successfully implemented, can provide valuable insights for household adoption.

*Adoption of AI in food waste management*: AI technologies, such as smart refrigerators, food tracking apps, and AI-powered supply chain systems, have been widely adopted in hospitality to optimize food consumption and reduce waste. For instance, restaurants use AI to predict customer demand and adjust inventory accordingly, reducing excess purchases and spoilage (Filimonau and Gherbin, 2017). The same principles can be applied in households, where AI systems help families track food expiration dates, monitor inventory, and reduce overbuying (van der Werf et al., 2021). However, adopting AI in households is slower due to several managerial challenges, including cost, accessibility, and consumer engagement. AI tools often deliver immediate financial benefits in commercial settings by reducing waste and optimizing operations. The financial return on investment is less obvious in households, which can deter adoption (Stancu et al., 2016). *Cost and accessibility*: One of the primary challenges in adopting AI-driven solutions in households is the cost of smart appliances and AI-driven systems. In the hospitality industry, large-scale operations justify the expense of AI technologies due to their ability to save costs by reducing waste (Principato et al., 2021). For individual households, however, the upfront cost of smart refrigerators, for example, can be prohibitively high.

Moreover, households may be unwilling to invest in technology if they do not perceive a clear financial benefit or if they lack familiarity with AI systems (Quested et al., 2013). Low-cost alternatives, such as food consumption tracking mobile apps, offer a more accessible option for households. Apps like OLIO (Olio, 2024) and Too Good To Go (Too Good To Go, 2024) allow users to monitor their food usage without needing expensive appliances. However, the long-term success of these tools depends on sustained engagement and regular use (Filimonau and Gherbin, 2017). *Technological infrastructure*: AI technologies require reliable data inputs and a robust technological infrastructure to function effectively. In hospitality, AI technological systems are supported by integrated supply chains and real-time data collection, which ensures that AI-driven recommendations are accurate and timely (Filimonau and Gherbin, 2017). In households, the lack of such infrastructure can limit the effectiveness of AI tools. For example, smart refrigerators and food tracking apps rely on users to input accurate food purchases and consumption data. Without consistent data, AI systems may struggle to provide useful recommendations, reducing their effectiveness (van der Werf et al., 2021). To overcome this barrier, AI systems for households must be highly automated and user-friendly, requiring minimal user input to maintain accurate data on food inventories and expiration dates (Roodhuyzen et al., 2017). *Consumer trust and data privacy*: Consumer trust is another significant barrier to adopting AI-driven technologies in household food waste management. Many consumers are wary of sharing personal data with AI systems, particularly when it comes to sensitive information about their purchasing habits and consumption patterns (Filimonau and Gherbin, 2017). In the hospitality industry, where data is collected and managed within a controlled environment, concerns about data privacy are less prevalent. To increase adoption in households, AI developers must prioritize transparency and address concerns about data privacy. Providing clear information about how data is used and the benefits of AI systems can help build consumer trust and encourage more widespread adoption (Quested et al., 2013).

While theoretical frameworks such as the Theory of Planned Behavior and Nudge Theory provide insights into how AI can influence household behavior, practical barriers such as cost, technological infrastructure, and consumer trust remain significant obstacles. By learning from the successes of AI-driven solutions in the hospitality industry and addressing these challenges, AI systems have the potential to transform household food management and significantly reduce food waste. Table 2 summarizes fundamental studies that span theoretical and managerial perspectives, offering insights into the behavioral theories that underpin consumer decision-making and the practical challenges associated with adopting AI technologies in households.

**Table 2:** Summary of references, study focus, key findings, and relevance to AI technology.

References	Focus of study	Key findings	Relevance to AI technology
Ajzen (1991)	Introduces the theory of planned behavior as a framework for behavior change relevant to understanding food waste behaviors.	Perceived control over behavior can reduce food waste through better management practices.	Provides foundational insights into behavior change, applicable in AI algorithms for personalized waste management recommendations.
Aschemann-Witzel et al. (2015)	Examines causes of consumer food waste and proposes potential interventions for behavior change.	Consumer education and awareness are critical for reducing food waste; identified psychological and societal causes.	Highlights behavioral causes of waste, which AI systems could use to customize interventions and awareness prompts.
Becker (1976)	Applies Rational Choice Theory to consumer decision-making in areas such as food waste management.	Perceived costs and benefits of waste reduction influence decision-making processes in food management.	Rational Choice Theory can be integrated into AI-driven decision-support systems for optimizing household purchasing behaviors.
Borrello et al. (2016)	Explores challenges in transitioning to a bio-based circular economy within the agri-food sector.	Identifies regulatory, economic, and technological challenges in reducing waste within a bio-based economy.	Identifies systemic challenges in food waste, which AI could help address by integrating supply chain and consumption data.
Bux (2024)	Investigates digital and conventional methods for monitoring food waste in healthcare settings.	Found digital solutions that are more accurate in waste tracking, providing better data for managing food supplies.	Digital methods for waste tracking align with AI’s role in data collection, analysis, and real-time monitoring.
Bux and Amicarelli (2024)	Discusses the role of blockchain in tracking food waste and enhancing sustainability in food security.	Blockchain can improve transparency in food waste monitoring, potentially increasing consumer trust in the food supply.	Shows blockchain potential for AI systems to enhance traceability and transparency in food waste management.
Filimonau and Gherbin (2017)	Examines food waste management practices in the UK grocery sector, focusing on sustainable practices.	Adopting sustainable waste practices can lead to economic and environmental benefits for grocery retailers.	Insights into retail practices can guide AI systems in optimizing inventory and reducing waste in household and commercial settings.
Principato et al. (2021)	Provides a systematic review of consumer behavior related to household food waste.	Consumers’ food waste behaviors are complex and shaped by social norms, habits, and situational factors.	The framework can support AI in identifying patterns and predicting high-risk waste behaviors.
Quested et al. (2013)	Analyzes complex behavioral patterns related to food waste using qualitative approaches.	Food waste behaviors are multifaceted; qualitative methods can capture the range of motivations behind waste.	Complex behavior analysis supports AI in designing adaptive feedback for user behavior improvement.
Roodhuyzen et al. (2017)	Proposes an integral perspective on consumer food waste behaviors to address waste issues holistically.	Suggests a holistic approach to waste management, integrating various consumer behavior perspectives.	Integral perspective aids AI in developing holistic waste management models, incorporating multiple factors.
Stancu et al. (2016)	Identifies key determinants of food waste behavior, focusing on psychological and habitual factors.	Found that psychological factors and daily habits significantly influence waste behavior in households.	Behavioral insights support AI in tailoring nudges and feedback loops to reduce household food waste.
Thaler and Sunstein (2008)	Nudge Theory as a framework for behavior influence, with applications for reducing food waste.	Nudge strategies can reduce waste by subtly guiding consumer choices without restricting options.	Nudge Theory is highly relevant for AI-based reminders and prompts encouraging sustainable food practices.
van der Werf et al. (2021)	Tests an intervention aimed at reducing household food waste through behavioral change techniques.	Behavioral interventions showed significant potential in reducing waste and increasing savings in households.	Behavioral intervention data supports AI models in designing household cost-saving and waste-reducing strategies.

This table provides a selective overview of key studies relevant to AI-driven food waste management and consumer behavior.

## 4 Methods and procedures

This study employed an instrumental case study approach (Creswell and Poth, 2016) to explore the potential applicability of AI-driven food waste management methods from the hospitality industry to the household environment (Creswell and Poth, 2016). The central focus of this case study is to identify and analyze AI-driven food waste management methods that have been successfully implemented in the hospitality industry, intending to evaluate their potential for adaptation in households. The selected case units for this study are three leading companies in the hospitality industry, i.e., Leanpath (Food Waste Intelligence, 2024), Winnow (The Food Waste Blog, 2024), and Kitro (Kitro, 2024). Each company has established a reputation for implementing advanced AI technologies in various hospitality settings, including hotels and restaurants. These companies utilize kitchen-ready trackers, integrated scales, and touchscreens as part of their AI-driven systems to monitor, track, and measure food waste.

The data collection was conducted through semi-structured interviews with key representatives from each selected company. Semi-structured interviews are an important source of case study evidence (Yin, 2017). The interviews were designed to gather comprehensive insights into the specific AI-driven technologies used by these companies, the challenges they encountered during implementation, and the overall impact of their initiatives on reducing food waste. In addition to exploring the technical aspects of the AI systems, the interviews also explore methods employed by these companies, such as machine learning algorithms, sensor technologies, and data analytics tools, to enhance the effectiveness of their food waste management strategies.

A thorough analysis of the recorded interview data was conducted to identify the core AI-driven methods and technologies utilized in the hospitality industry and to evaluate their potential for application in household settings. This analysis involved transcribing the interview data and systematically categorizing the identified technologies based on their specific applications in reducing food waste. The process included (a) identifying AI-driven technologies, such as machine learning algorithms, sensor technologies, and data analytics tools, and categorizing these technologies based on their role in reducing food waste. Additionally, we analyzed the methods the hospitality industry uses to integrate AI into food waste management practices, focusing on the strategies and techniques for collecting and analyzing data related to food waste. The analysis also involved (b) identifying the challenges and benefits of AI-driven food waste management in the hospitality industry, particularly focusing on practical considerations and obstacles when implementing similar solutions in household settings. The transcribed data were then sent to the interviewed representatives from each company for peer review, with the understanding that certain proprietary protocols and sensitive information could not be disclosed or shared publicly. This step ensured the accuracy of the information while maintaining the confidentiality of company-specific practices.

The study also aimed to assess the feasibility of adapting these AI-driven technologies for use in household settings, which involved evaluating the practical considerations, challenges, and potential benefits of implementing similar solutions in a domestic environment. Key factors considered in this evaluation included the variability in food consumption patterns in households compared to hospitality settings, the economic viability of such technologies for individual consumers, and the potential obstacles that might arise during the adaptation process.

In summary, this comprehensive approach combines detailed data collection with in-depth analysis, providing a clear pathway for assessing the potential for AI-driven food waste management technologies to be adapted from the hospitality industry to the household environment. By systematically exploring the methods, technologies, and practical implications of such an adaptation, the study offers valuable insights into the broader application of AI in promoting sustainable food waste management practices across different contexts.

### 4.1 The case

This research examines the application of AI-driven food waste management practices by three leading companies servicing the hospitality industry: Leanpath, Winnow, and Kitro. These companies, serving as the case study’s units, operate within the food service sector, targeting hotels, restaurants, and institutional cafeterias, including those in colleges, hospitals, corporate settings, sports, and leisure facilities. These companies primarily focus on the commercial market. Their platforms for preventing food waste incorporate kitchen-ready trackers, integrated scales, and touchscreens, allowing for efficient tracking, measurement, and photographic documentation of food waste. These platforms process data through a sophisticated analytical platform, providing insights into the causes of waste generation and encouraging behavioral change.

This study examines companies using AI-driven technologies in commercial kitchens to monitor and reduce food waste. The study examines machine learning algorithms, sensor technologies, and data analytics tools employed within the hospitality industry to identify, categorize, and quantify food waste and generate actionable insights for reduction. The study also considers the AI-driven methods and approaches the hospitality industry employs to integrate AI into food waste management processes, including strategies and techniques for data collection and analysis related to food waste. Additionally, the study assesses the challenges and benefits of adopting AI in the hospitality sector, identifying obstacles to implementing AI-driven food waste solutions and the advantages of their use.

## 5 Findings

**Figure 1:**
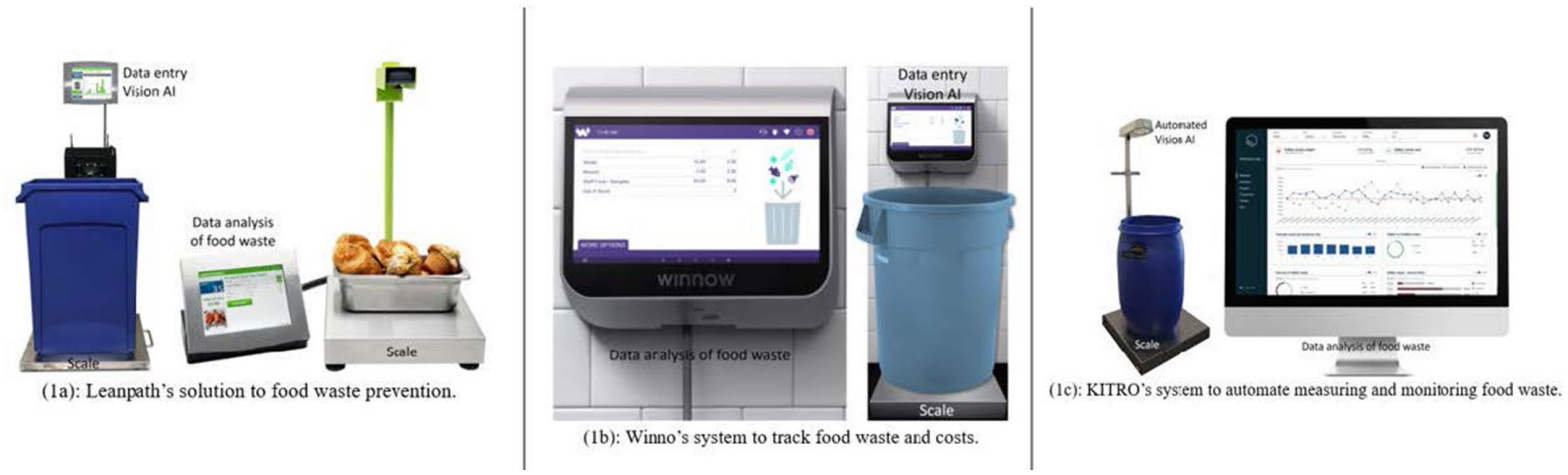
**(1a)** Leanpath, **(1b)** Winnow, and **(1c)** Kitro’s commercially available models for AI-driven food waste management systems as of 2024.

Findings reveal Leanpath, Winnow, and Kitro’s effectiveness in employing AI-driven food waste management systems in hospitality. The analysis covers their technological solutions, system configurations, data analytics, and user engagement, discussing the potential for home use and impacts on sustainability and efficiency. The industry term for these AI-driven bins is smart-bin. Figure 1 illustrates a smart-bin setup of (1a) Leanpath, (1b) Winnow, and (1c) Kitro’s commercially available models for AI-driven food waste management systems as of 2024.

### 5.1 Leanpath

Leanpath was founded in 2004, creating the first automated food waste measurement technology system for food service and hospitality kitchens and, in the process, creating an industry. Their system integrates a touchscreen terminal with scales and cameras and AI recognition technology across a suite of models, capturing critical data such as the type and amount of food waste per transaction, the cost, the reason for the waste, the source, and the disposition of the waste. The data (and digital images on specific models) flow to Leanpath’s cloud-based platform (Leanpath Online) for analysis of the composition of food waste across multiple dashboards coupled with behavior change functionality, which leverages AI and culinary experts to guide operational changes to prevent the recurrence of food waste.

#### AI-driven methods and approaches employed:

Leanpath utilizes a “track-discover-drive” methodology. When kitchen staff discard food, the type of food and the reason for discarding it (e.g., overproduction, spoilage, trim waste) are tracked in seconds on a touchscreen terminal that draws on a customized menu specific to each location. A comprehensive analytics platform complements this process: multiple dashboards with intuitive graphics, a digital photo stream, and alert functionality to flag specific waste transactions in real-time interventions, goal functionality to target and track waste reduction for specific high-value items over a defined period, donation tracking, and AI-enhanced coaching tips. The system provides comprehensive reports (at individual and enterprise levels) to identify types and sources of food waste, enabling establishments to take actionable steps like adjusting batch sizes or modifying menus and portion sizes. Leanpath also emphasizes staff engagement and continuous monitoring to cultivate a culture of food waste prevention, including a leaderboard feature to encourage participation and positive competition between locations. They offer coaching by experienced chefs to integrate food waste prevention techniques into daily kitchen practices.

#### Challenges and benefits faced:

In Leanpath’s startup phase, Leanpath faced typical logistical and cultural challenges of scaling globally and educating busy food service leaders on the importance of committing to a bold food waste reduction goal and the strong value proposition within food waste prevention. Despite these challenges, Leanpath has made notable achievements in food waste reduction, typically cutting food waste by 50% in client sites and reducing food purchase costs by up to 6% while engaging frontline food service workers in creating a culture of food waste prevention. Today, the company operates in over 4,000 locations and 29 languages across 45 countries. It has prevented over 120 million pounds of food waste since its inception. In addition to cost savings, Leanpath’s platform provides environmental data associated with food waste savings for sustainability reporting.

### 5.2 Winnow

Winnow was established in 2013. They introduced their AI model Winnow Vision Technology in 2019, significantly impacting food waste management in the hospitality industry. This system comprises a scale and a smart camera positioned above a standard waste bin, capturing images and data of discarded food in real-time. The Vision box, equipped with a light and motion-sensitive camera, identifies various food items as they are thrown away. A touchscreen tablet facilitates staff interaction when required and displays immediate feedback on the most recent food waste transaction’s financial and environmental impact.

#### AI-driven methods and approaches employed:

Winnow’s AI capability works in real-time and is enabled on day one of usage within a client kitchen. The staff may encounter two scenarios on the touchscreen when food is scraped into the bin, depending on the confidence level of the AI for the item that was thrown away. When the AI has reached a certain threshold of confidence in identifying the item, the system will automatically categorize the food waste transaction without any screen interaction required from the staff. In cases where the AI’s confidence has not reached the threshold required for automatic, no-touch categorization, the system will predict a shortlist of eight potential items -- these eight menu items are then displayed on the screen for the staff to choose from. Each instance in which the staff provides the system with the correct selection helps inform the universal AI model for the next time that item is thrown away to reach automatic categorization with most of a kitchen’s food waste items. Winnow also provides clients with leading analytics. This includes periodic automated PDF reports and access to their online dashboard, Winnow Hub, which allows users with plenty of functionality to view and manipulate the food waste data in real-time, download additional reports, and compare performance across locations in the case of a multi-site client. Winnow also provides a dedicated customer success manager for each new client, who provides the staff with coaching to discuss the reports and help identify reduction opportunities within the data.

#### Challenges and benefits faced:

Winnow faced several challenges, including bin placement issues, security concerns during installations, and the need for staff engagement. Resistance from staff not using the system correctly or prioritizing waste reduction also posed obstacles. However, the benefits have been significant. Winnow’s approach has resulted in an average of 53% reduction in food waste across their customer base, resulting in substantial economic savings. This achievement aligns with a shift in client focus from cost savings to sustainability goals.

### 5.3 Kitro

Established in 2017, Kitro’s AI-driven technology comprises a camera and weighing scale system designed to automatically detect and categorize food waste. The system utilizes weight sensors and cameras to capture images of discarded food, which are then processed using deep learning algorithms. This technology is deployed in various settings, including commercial kitchens, buffets, and guest plates, to monitor food waste effectively.

#### AI-driven methods and approaches employed:

Kitro employs a centralized algorithm that works across all properties, negating the need for individual menu data. The system records the waste’s weight, timing, and content when food is discarded. Image recognition and categorization of items occur in the cloud, where Kitro’s algorithm recognizes approximately 300+ food items. This granularity enables detailed data analysis. Kitro’s dashboard allows clients to filter data by categories, service times, and reasons for waste, assigning a price to each ingredient to calculate total food waste value.

#### Challenges and benefits faced:

Potential clients were initially reluctant to invest due to the perceived slowing of productivity caused by manual data entry. However, these obstacles have been overcome by Kitro, which offers 100% automation for customers where no manual input or labeling is required, leading to significant benefits. Kitro’s clients report an average reduction in food waste of 30%, with some seeing a decrease of over 50%. The system has also led to substantial cost savings, with average annual savings of 2 to 8% of their annual food costs, demonstrating a solid return on investment.

In summary, Leanpath, Winnow, and Kitro offer innovative AI-driven food waste management solutions for the hospitality industry, each employing different technologies tailored to their unique approaches: Leanpath uses AI-powered cameras and scales to track food waste in real-time, capturing images and data that feed into a cloud-based dashboard to identify waste patterns and inform reduction strategies. Winnow integrates AI with cameras and scales to automatically identify discarded food items and quantify waste, providing actionable insights that allow kitchens to optimize inventory and reduce waste costs. Kitro offers a fully automated solution using sensors and cameras to record waste without manual input, analyzing the data to help businesses enhance sustainability and cut costs. Table 3 provides a detailed comparison of these companies’ technologies, methods, and the potential benefits for household adaptation.

**Table 3:** Comparison of AI-driven food waste management technologies in the hospitality industry.

Company	Core technology	AI-driven methods	Waste measurement technique	Potential household benefits	Scope of company efforts
Leanpath, est. 2004	Integrated system with touchscreen, scales, cameras, and AI recognition	*Track-Discover-Drive* methodology: real-time tracking, customizable dashboards, behavior change functionality, AI-enhanced coaching	Tracks type, amount, reason, source, and disposal of food waste per transaction	Detailed tracking, actionable insights, staff engagement, continuous monitoring	Prevented 120 M+ lbs. of food waste, operating in 4,000+ locations across 45 countries
Winnow, est. 2013	Smart camera, scale, and touchscreen system with AI for real-time tracking	*Winnow Vision Technology*: AI categorizes waste automatically or suggests items for staff input; provides analytics via Winnow Hub and periodic PDF reports	Real-time categorization with financial and environmental impact feedback	Immediate feedback, easy categorization, staff training, and engagement	Launched AI model in 2019, 53% average reduction in food waste
Kitro, est. 2017	Camera and weighing scale system with image recognition	Centralized AI model: automatic detection and categorization, cloud-based processing, dashboards for analysis and reporting	Records weight, timing, and content; uses image recognition for categorization	Fully automated, no manual input, cost savings, detailed analysis	Founded in 2017, achieves a 30% average waste reduction, up to 50% in some cases

AI-driven refers to artificial intelligence technology.

## 6 Discussion

The innovative smart-bin products from companies like Leanpath, Winnow, and Kitro utilize AI-driven technologies to address food waste in the hospitality industry. These systems combine AI imaging recognition and weighing mechanisms to monitor and quantify food waste, focusing on sustainability and cost savings. Smart bins are designed to identify the types of food wasted and measure their weight. Once quantified, the waste is translated into financial losses and associated greenhouse gas emissions. The data collected is integral to the technical development of these systems, informing the algorithmic processes and the detailed data provided to customers. These systems face challenges, including scalability, cultural and language barriers in global markets, and logistical complexities of managing hardware components. Customer support is pivotal for effective implementation, with companies offering training and continuous monitoring to encourage staff engagement in waste reduction practices. The pricing models and return on investment (ROI) are significant factors, with some clients reporting substantial cost savings. Looking ahead, these companies are committed to continuous innovation, aiming to enhance their products’ accuracy while exploring adaptations for potential household use to further contribute to global waste reduction efforts.

The smart-bin products from Leanpath, Winnow, and Kitro each offer custom approaches to using AI-driven technology to reduce food waste in the hospitality industry. Leanpath’s system focuses on continuous monitoring and detailed data analysis, identifying patterns in food waste, and developing actionable waste minimization strategies while continually reporting progress against an established baseline. Winnow utilizes a system that evolved from manual logging to AI-driven technology. Winnow’s Vision system features a camera and scale setup to capture real-time images and data of discarded food. The system predicts the type of waste using a universal model and automates the categorization process over time. Winnow’s approach includes detailed client reports and online data analysis tools, with a notable shift toward sustainability goals among their clientele. Kitro, on the other hand, employs a scale and a camera to capture the weight and images of discarded food, which is processed by deep learning algorithms. Kitro’s system offers a centralized algorithm that eliminates the need for individual menu data and recognizes approximately 300+ food items. This system provides extensive data analysis capabilities and client insights, assisting in optimizing operational processes. Leanpath adopts a “track-discover-drive” methodology, combining a touchscreen terminal with a scale and supplementing it with manual photo analysis and AI recognition. This approach emphasizes the tangible (and repetitive) nature of food waste and engagement with frontline food service workers, coupled with detailed waste reduction resources, behavior change functionality, and expert coaching from veteran culinary professionals to foster a waste-conscious culture.

## 7 Potential implications

### 7.1 Adaptation for household use

The transition from manual to automated food waste management processes, facilitated by AI-driven technologies, highlights the potential for significant environmental and economic benefits in commercial and residential settings. This approach should consider households’ specific needs and behaviors and the technological, economic, and educational factors that influence the adoption and effectiveness of AI-driven solutions.

#### 7.1.1 Lessons from the hospitality industry

The successful application of AI-driven technologies by companies such as Leanpath, Winnow, and Kitro in the hospitality industry provides valuable insights for potential household adaptation. These systems have significantly reduced food waste in commercial settings through continuous monitoring, real-time data analytics, and user engagement. For example, Leanpath’s systems have reduced food waste by up to 50% in some settings by providing detailed feedback on waste generation and offering actionable insights for reduction (Food Waste Intelligence, 2024). Similarly, Winnow’s AI technology has achieved an average 53% reduction in food waste through real-time categorization and feedback (The Food Waste Blog, 2024; Nu et al., 2024). These successes suggest that, with proper adaptation, similar outcomes could be achieved in households.

#### 7.1.2 Practical considerations for household adaptation

Adapting AI-driven technologies for residential use involves addressing several key challenges. First, the scale and complexity of the systems used in commercial kitchens must be reduced to suit the household environment. This includes developing compact, user-friendly devices that integrate seamlessly into daily household routines. For instance, Leanpath’s technology could be miniaturized to fit under kitchen counters, with simplified interfaces allowing users to track and analyze their food waste.

Moreover, continuous monitoring and real-time feedback, which are central to the effectiveness of these systems, must be tailored to the more variable and less predictable nature of household food consumption. Studies have shown that household food waste is often driven by over-purchasing, improper storage, and lack of meal planning (Parizeau et al., 2015). AI-driven systems could address these issues by providing personalized recommendations based on past behavior, suggesting optimal portion sizes, and offering reminders to use perishable items before they spoil.

#### 7.1.3 Policy support and educational initiatives

Supportive policies and educational initiatives are crucial for adopting AI-driven food waste management technologies that are widely adopted in households. Research has shown that public awareness and education play significant roles in adopting sustainable practices (Moser, 2016). Governments and organizations could promote AI technologies through subsidies, incentives, and public campaigns highlighting their benefits in reducing food waste and saving money. Additionally, online platforms could offer training modules similar to those provided by Winnow in the hospitality industry but tailored to the needs of household users.

#### 7.1.4 Scientific evaluation of household implementation

A rigorous scientific evaluation must be conducted to ensure the successful adaptation of these technologies. Pilot programs in diverse household settings could be used to test the effectiveness of these AI-driven systems, gathering data on food waste reduction, user engagement, and cost savings. These studies should also consider the long-term sustainability of AI integration in households, exploring factors such as energy consumption, data privacy, and system maintenance. For example, Kitro’s focus on continuous system improvement could be applied in the household context by regularly updating AI algorithms based on user feedback and waste patterns to maintain accuracy and relevance.

#### 7.1.5 Integration with smart home systems

Finally, integrating AI-driven food waste management technologies with existing smart home systems could enhance their functionality and ease of use. By connecting with smart refrigerators, voice-activated assistants, and mobile apps, these systems could provide holistic food management solutions that help households reduce waste while optimizing grocery shopping and meal planning. For example, sensors in smart refrigerators could alert users when items are nearing their expiration date. At the same time, AI-driven apps could suggest recipes that use up leftover ingredients, further reducing waste.

### 7.2 Policy recommendations and implementation

A multifaceted strategy encompassing policy recommendations and implementation steps is essential to harness the full potential of AI-driven technologies in household food waste management. Such a strategy should facilitate adopting and integrating these technologies into daily life and address critical interoperability, privacy, economic viability, and public engagement challenges. By developing comprehensive policies and initiatives, stakeholders can ensure that AI-driven solutions for food waste management are accessible, secure, and practical, contributing to environmental sustainability and household economic savings. The following strategies outline the critical components of such an approach, aiming to create a robust framework for the widespread adoption of AI in reducing food waste at the household level.

#### 7.2.1 Establishing standardized protocols and frameworks

Developing and enforcing standardized protocols and frameworks is crucial for ensuring interoperability among AI systems in food waste management. This standardization enables seamless interaction and efficient data sharing between AI tools and platforms, facilitating a unified approach to tracking and analyzing food waste patterns. Additionally, standardization can accelerate the adoption of AI-driven technologies by providing a clear set of guidelines for developers and users.

#### 7.2.2 Ensuring robust data privacy and security

Implementing stringent data privacy and security measures is essential in protecting sensitive household information collected by AI-driven food waste management systems. This includes employing advanced encryption methods, secure data storage solutions, and strict access controls to prevent unauthorized data access and breaches. Protecting personal data upholds user confidentiality and builds trust in AI technologies, encouraging wider acceptance and use.

#### 7.2.3 Providing economic incentives

Offering economic incentives, such as tax breaks, subsidies for purchasing smart appliances equipped with AI-driven technologies, and rewards for verifiable reductions in food waste, can motivate households to invest in and adopt AI-driven solutions. These incentives can lower the financial barriers to acquiring new technologies and highlight the economic benefits of reducing food waste, making adopting sustainable practices more attractive to consumers.

#### 7.2.4 Facilitating collaboration with technology providers

Forming strategic partnerships with technology providers and appliance manufacturers can ease the integration of AI tools into household appliances and enhance the development of innovations tailored to specific user needs. Collaborations can leverage the strengths of both the public and private sectors, driving the creation of user-friendly, efficient, and practical solutions for managing food waste at the household level.

#### 7.2.5 Launching public awareness initiatives

Raising public awareness about the importance and benefits of AI-driven food waste management is key to fostering cultural and behavioral change. This can be achieved through comprehensive educational programs, food waste management topics integration into school curricula, and national media campaigns designed to inform and engage the public. Highlighting food waste’s environmental and economic impacts can galvanize individuals and communities to adopt more sustainable practices.

#### 7.2.6 Implementing monitoring and feedback mechanisms

Establishing robust monitoring and feedback mechanisms allows for the continuous refinement of AI tools and a deeper understanding of user behavior and preferences. Collecting and analyzing data on how households use AI-driven systems for food waste management can inform future improvements and innovations, ensuring these solutions remain effective and user-friendly.

Integrating AI-driven technologies into household food waste management will likely require a concerted effort that spans policy formulation, technological innovation, economic incentives, and public engagement. By establishing standardized protocols, ensuring data privacy and security, offering economic incentives, fostering collaborations with technology providers, launching public awareness initiatives, and implementing monitoring and feedback mechanisms, we can create a supportive ecosystem for AI technologies to thrive. This comprehensive approach addresses the immediate challenges of adopting AI for food waste management and sets a foundation for sustainable practices that can lead to significant environmental and economic benefits. As we move forward, it will be crucial to continuously evaluate and adapt these strategies to meet evolving needs and maximize the positive impact of AI on reducing food waste in households.

### 7.3 Research and educational strategies

A detailed focus on research and educational strategies is paramount to optimizing the implementation of AI-driven technologies and their efficacy in household food waste management. These strategies should aim to advance the technological aspects of AI solutions and address the human, ethical, and economic factors influencing their adoption and impact (The Food Waste Blog, 2024). The following strategies outline a framework for integrating research and education into developing and disseminating AI-driven food waste management solutions.

#### 7.3.1 Behavioral studies on food consumption and waste

Conducting in-depth studies to understand the underlying human behaviors contributing to food consumption and waste is essential. These studies should examine psychological, cultural, and socioeconomic factors influencing household food waste behaviors. Insights gained from such research can inform the design of targeted educational interventions, promoting sustainable food consumption and waste reduction practices.

#### 7.3.2 Addressing ethical concerns in AI usage

Future research must tackle ethical issues, especially data privacy and security in household AI applications. Raising awareness about these ethical considerations through educational campaigns and discussions can encourage the responsible use of AI-driven technologies and ensure that privacy concerns do not hinder adoption.

#### 7.3.3 Fostering cross-sector partnerships

Establishing collaborations among AI developers, waste management organizations, and governmental agencies is crucial for developing comprehensive and practical solutions. Educational programs can serve as a bridge for these partnerships, offering forums for knowledge sharing, discussion, and collaborative innovation. Such collaborations can also leverage diverse expertise to tackle the multifaceted challenges of food waste management.

#### 7.3.4 Economic impact studies

Undertaking thorough studies to assess the economic impacts of AI-driven food waste management systems is vital. These studies should analyze cost savings, potential revenue streams, and broader economic benefits of reducing food waste. Developing educational content based on these findings can help households understand the financial advantages of adopting AI solutions, enhancing stakeholder engagement and support.

#### 7.3.5 Sustainability and technological evolution

Evaluating AI solutions’ long-term sustainability and adaptability within the rapidly evolving technological landscape is critical. Research in this area should focus on the durability of AI-driven technologies, their adaptability to new environmental challenges, and their compatibility with future technological advancements. Educational efforts can emphasize the importance of sustainable practices and the role of AI in promoting environmental stewardship.

#### 7.3.6 Integrating AI into educational programs

Investigating how AI technologies can be incorporated into educational curricula and programs is necessary to increase awareness and understanding of food waste management challenges and solutions. Initiatives could include developing workshops, seminars, and educational materials tailored to students and the broader community. We can foster a more sustainable future by educating younger generations and engaging them in AI-driven food waste management efforts.

Integrating AI-driven technologies into household food waste management necessitates a comprehensive approach combining in-depth research with targeted educational initiatives. By understanding and addressing the complex interplay of human behaviors, ethical considerations, economic impacts, and technological evolution, we can enhance the adoption and effectiveness of these solutions. The strategies outlined above highlight the importance of behavioral studies, ethical research, cross-sector partnerships, economic impact analyses, sustainability evaluations, and educational program integration. Together, these elements form a holistic framework for advancing AI-driven food waste management in a way that is expected to be both impactful and sustainable. Moving forward, it will be essential to continue exploring these areas, fostering collaboration and innovation to reduce food waste and promote environmental stewardship at the household level.

## 8 Limitations

While this study provides valuable insights into the potential application of AI-driven food waste management strategies from the hospitality industry to household settings, several limitations must be recognized. The study focused on three leading companies, i.e., Leanpath, Winnow, and Kitro - within the hospitality industry. While these companies represent some of the most advanced AI-driven food waste management systems, the findings may not fully capture the diversity of approaches and technologies available in other sectors or smaller-scale operations. This limitation suggests the need for broader research encompassing a wider range of companies and industries. The study’s primary focus was on the hospitality industry, which operates on a different scale and with various economic incentives compared to household environments. The variability in household food consumption patterns, waste generation, and technological adoption presents challenges that the study may not have fully addressed (Nunkoo et al., 2021). These differences might limit the direct applicability of the findings to the household context without further adaptation and testing. The study assumes a certain level of technological literacy and willingness to adopt AI-driven solutions among households. However, the readiness of average consumers to engage with these technologies may vary, affecting the feasibility of widespread adoption (Nunkoo et al., 2021; Van Geffen et al., 2020).

Additionally, the economic feasibility for households to invest in AI-driven systems may differ significantly from commercial entities, which could limit the potential impact of these technologies at the household level (Fang et al., 2023; Onyeaka et al., 2023). Implementing AI-driven technologies in households raises concerns about data privacy and security, which this study did not explore extensively. While the commercial sector may have more robust systems to protect sensitive data, households may face challenges ensuring their personal information is secure when using AI-driven food waste management systems (Anggraeni et al., 2021). Finally, the study’s findings are based on a specific set of AI-driven technologies and operational practices within the hospitality industry. As such, the generalizability of these findings to other contexts, such as smaller businesses, different cultural settings, or varying levels of technological infrastructure, may be limited. Further research is necessary to explore how these AI-driven solutions could be tailored to meet the unique needs of different user groups.

## 9 Conclusion

This paper explored the potential of AI-driven food waste management strategies used in the hospitality industry for applications to the typical household. Utilizing AI for household food waste management offers opportunities for research, innovation, and societal change. While this paper lays the groundwork for potential applications, the future of this promising domain will be shaped by the evolution of technology, policy, societal attitudes, and effective educational strategies. The urgent need to address food waste, with extensive environmental, economic, and social ramifications, underscores the importance of innovative solutions, particularly in household management. This exploration into the transformative potential of AI technologies, initially tailored for the hospitality industry, reveals a promising avenue for tackling food waste. The convergence of AI advancements, digital connectivity, and societal focus on sustainability presents a unique opportunity. The proposed framework, including standardized AI solutions, robust data privacy protocols, economic incentives, public awareness initiatives, and collaboration with technology providers, offers a comprehensive approach to harnessing AI’s capabilities for sustainable food waste management. While challenges in implementation are acknowledged, the pathway illuminated by these innovations, supported by informed policies and societal engagement, holds promise for significant waste reduction and cultivating a more sustainable global community. The study proposes avenues for adapting these technologies for household use, potentially offering scalable solutions to global food waste challenges. The adaptability of these systems to home environments hinges on simplifying the technology for personal use while retaining the core functionalities that facilitate waste awareness and reduction. Exploring these systems’ potential adaptation to household settings opens exciting possibilities for reducing global food waste, enhancing sustainability, and driving economic savings. These companies’ AI-driven food waste management practices offer valuable lessons for the hospitality industry and signal broader applicability to households and potentially other commercial settings.

## Data Availability

The data supporting this study’s findings are not publicly available due to confidentiality agreements/legal restrictions, further inquiries can be directed to the corresponding author.
